# Supplementary material for: Flagellar rotation facilitates the transfer of a bacterial conjugative plasmid
Source: EMBO J. 2024 Dec 2;44(2):587–611. doi: 10.1038/s44318-024-00320-0 (PMC11730352; doi:10.1038/s44318-024-00320-0)
Supplement: Supplementary file 1 — Table EV1 [file 44318_2024_320_MOESM1_ESM.docx]

| **Strain name**  **Table EV1: List of bacterial strains used in this study** | **Genotype** | **Strain construction** |
| --- | --- | --- |
| PY79 | *B. subtilis* wild type | (Youngman *et al*, 1984) |
| GB61 | ∆*ymdB::tet* | (Dubey *et al*, 2016) |
| GD215 | ∆*hag::erm* | (Dubey & Ben-Yehuda, 2011) |
| SB463 | *amyE::*P*_hyper-spank_*-*cat-spec-lacI* | (Dubey & Ben-Yehuda, 2011) |
| SB513 | *amyE::*P*_hyper-spank_*-*gfp-kan* | (Dubey & Ben-Yehuda, 2011) |
| AR16 | *amyE::*P*_rrnE_*-*gfp-spec* | (Rosenberg *et al*, 2012) |
| ET13 | *amyE::*P*_hyper_*_-_*_spank_*-*yueB*-*yfp*-*spec* | (Tzipilevich *et al*, 2017) |
| IB97 | ∆*sigD::tet* | Laboratory stock |
| GD127 | *B. subtilis* 168/pLS20_cm_ | Kindly provided by Prof. Mitsuhiro Itaya (Keio U) (Dubey & Ben-Yehuda, 2011) |
| DS1895 | *amyE::*P*_hag_-hag*^T209C^-*spec* | Kindly provided by Prof. Daniel B. Kearns (Indiana U) |
| BDR2637 | *sacA::*P*_veg_-mCherry*-*phleo* | Kindly provided by Prof. David Rudner (Harvard U) |
| BKK13690 | ∆*motA::kan* | *B. subtilis* deletion Library (Koo *et al*, 2017) |
| BKK35430 | ∆*flgM::kan* | *B. subtilis* deletion Library (Koo *et al*, 2017) |
| BKK35490 | ∆*degU::kan* | *B. subtilis* deletion Library (Koo *et al*, 2017) |
| PK143 | ∆*sigD::tet*/pLS20_cm_ | pLS20_cm_ was introduced into IB97 by conjugation with SH337. |
| GV279 | ∆*fliW::erm* | Constructed by Gibson master mix to assemble PCR products, amplified from PY79 genomic DNA, using primers 5122-5123, 5124-5125, and from pWX467 using primers 2430-2431. The mixture was transformed into PY79. |
| GV280 | ∆*csrA::erm* | Constructed by Gibson master mix to assemble PCR products, amplified from PY79 genomic DNA, using primers 5126-5127, 5128-5129, and from pWX467 using primers 2430-2431. The mixture was transformed into PY79. |
| GV281 | ∆*fliW-hag::erm* | Constructed by Gibson master mix to assemble PCR products, amplified from PY79 genomic DNA, using primers 5122-5123, 5130-5131, and from pWX467 using primers 2430-2431. The mixture was transformed into PY79. |
| GV283 | ∆*csrA-hag::erm* | Constructed by Gibson master mix to assemble PCR products, amplified from PY79 genomic DNA, using primers 5126-5127, 5130-5131, and from pWX467 using primers 2430-2431. The mixture was transformed into PY79. |
| GV289 | ∆*fliW::erm/*pLS20_cm_ | pLS20_cm_ was introduced into GV279 by conjugation with SH337. |
| GV290 | ∆*csrA::erm/*pLS20_cm_ | pLS20_cm_ was introduced into GV280 by conjugation with SH337. |
| GV291 | ∆*fliW-hag::erm/*pLS20_cm_ | pLS20_cm_ was introduced into GV281 by conjugation with SH337. |
| GV293 | ∆*csrA-hag::erm/*pLS20_cm_ | pLS20_cm_ was introduced into GV283 by conjugation with SH337. |
| MBS7 | *amyE::*P*_rrnE_*-*gfp-spec*/pLS20_cm_ | pLS20_cm_ was introduced into AR16 by conjugation with GD127. |
| MBS11 | ∆*hag::erm,* *amyE::*P*_hyper-spank_*-*gfp-Kan* | SB513 was transformed with GD215 genomic DNA. |
| MBS17 | ∆*hag::erm*/ pLS20_spec_-P*_hyper-spank_*-*gfp* | pLS20_spec_-P*_hyper-spank_*-*gfp* was introduced into GD215 by conjugation with SH342. |
| MBS20 | ∆*hag::kan*/pLS20_cm_-*ssb-yfp-erm* | pLS20_cm_-*ssb-yfp-erm* was introduced into SH91 by conjugation with SH347. |
| MBS23 | ∆*ymdB::tet*/pLS20_cm_-*tie*_2xHA_-*erm* | pLS20_cm_-*tie*_2xHA_-*erm* was introduced into GB61 by conjugation with SH461. |
| MBS24 | ∆*hag::kan*/pLS20_cm_-*tie*_2xHA_-*erm* | pLS20_cm_-*tie*_2xHA_-*erm* was introduced into SH91 by conjugation with SH461. |
| MBS25 | ∆*sigD::tet*/pLS20_cm_-*tie*_2xHA_-*erm* | pLS20_cm_-*tie*_2xHA_-*erm* was introduced into IB97 by conjugation with SH461. |
| MBS26 | ∆*CORE* *(fliO-flhA)::tet,* P*_fla/che_-flhF*/pLS20_cm_-*tie*_2xHA_-*erm* | pLS20_cm_-*tie*_2xHA_-*erm* was introduced into SH9 by conjugation with SH461. |
| MR13 | WT/pLS20_cm_-*conAn1_-_*_2xHA_-*erm* | Constructed by Gibson master mix to assemble PCR products, amplified from MBS7 DNA, using primers 7054-7055, 7056-7057, and from SH347 DNA, using primers 5562-2431. The mixture was transformed into SH337. |
| LZ4 | ∆*hag::kan*/pLS20_cm_-*conAn1_-_*_2xHA_-*erm* | MR13 was transformed with SH91 genomic DNA. |
| LZ5 | ∆*CORE* *(fliO-flhA)::tet,* P*_fla/che_-flhF*/pLS20_cm_-*conAn1_-_*_2xHA_-*erm* | MR13 was transformed with SH9 genomic DNA. |
| LZ6 | ∆*sigD::tet*/pLS20_cm_-*conAn1_-_*_2xHA_- *erm* | MR13 was transformed with IB97 genomic DNA. |
| LZ7 | ∆*motA::kan*/pLS20_cm_-*conAn1_-_*_2xHA_-*erm* | MR13 was transformed with BKK13690 genomic DNA. |
| LZ8 | ∆*ymdB::tet*/pLS20_cm_-*conAn1_-_*_2xHA_-*erm* | MR13 was transformed with GB61 genomic DNA. |
| LZ9 | ∆*flgM::kan*/pLS20_cm_-*conAn*_2xHA_-*erm* | MR13 was transformed with BKK35430 genomic DNA. |
| LZ16 | ∆*flgM::kan*/pLS20_cm_-*tie*_2xHA_-*erm* | SH461 was transformed with BKK35430 genomic DNA. |
| LZ53 | *amyE::*P*_hyper-spank_*-*epsE*-*spec*/pLS20_cm_ | SH337 was transformed with pLZ3. |
| SH9 | ∆*CORE (fliO-flhA)::tet*, P*_fla/che_-flhF* | (Bhattacharya *et al*, 2019) |
| SH91 | ∆*hag::kan* | Constructed by Gibson master mix, to assemble PCR products, amplified from PY79 genomic DNA, using primers 2513-2514, 2516-2517, and from pWX470 using primers 2430-2431. The mixture was transformed into PY79. |
| SH101 | ∆*ymdB::tet*, *amyE::*P*_hag_-hag*^T209C^-*spec* | DS1895 was transformed with GB61 genomic DNA. |
| SH337 | WT/pLS20_cm_ | pLS20_cm_ was introduced into PY79 by conjugation with MBS7. |
| SH342 | WT/pLS20_spec_-P*_hyper-spank_*-*gfp* | Constructed by Gibson master mix, to assemble PCR products, amplified from MBS7 DNA, using primers 5141-5142, 5143-5144, and from pSH46 DNA using primers 5145-5146. The mixture was transformed into SH337. |
| SH345 | *sacA::kan* | Constructed by Gibson master mix, to assemble PCR products, amplified from PY79 genomic DNA, using primers 3334-5179, and 3336-3337, and from pWX470 using primers 2430-2431. The mixture was transformed into PY79. |
| SH347 | WT/pLS20_cm_-*ssb-yfp-erm* | Constructed by Gibson master mix, to assemble PCR products, amplified from MBS7 DNA, using primers 5171-5174, 5175-5176, and from ET13 genomic DNA, using primers 5172-3416, and from pWX467 using primers 2430-2431. The mixture was transformed into SH337. |
| SH352 | ∆*CORE (fliO-flhA)::tet*, P*_fla/che_-flhF*/pLS20_cm_ | pLS20_cm_ was introduced into SH9 by conjugation with SH337. |
| SH359 | WT/pLS20_spec_-P*_hyper-spank_*-*gfp, sacA::*P*_veg_-mCherry*-*phleo* | pLS20_spec_-P*_hyper-spank_*-*gfp* was introduced into BDR2637 by conjugation with SH342. |
| SH360 | *sacA::spec* | Constructed by Gibson master mix, to assemble PCR products, amplified from PY79 genomic DNA, using primers 3334-5179, and 3336-3337, and from pWX466 using primers 2430-2431. The mixture was transformed into PY79. |
| SH363 | WT/pLS20_spec_-P*_hyper-spank_*-*gfp, sacA::*P*_veg_-mCherry*-*phleo, amyE::*P*_hyper-spank_*-*cat-spec-lacI* | SH359 was transformed with SB463 genomic DNA. |
| SH368 | ∆*csrA::erm,* ∆*flgM*::*kan* | GV280 was transformed with BKK35430 genomic DNA. |
| SH381 | ∆*csrA::erm,* ∆*flgM::kan/*pLS20_cm_ | pLS20_cm_ was introduced into SH368 by conjugation with SH337. |
| SH408 | ∆*flgM::kan*, *amyE::*P*_hag_-hag*^T209C^-*spec* | DS1895 was transformed with BKK35430 genomic DNA. |
| SH409 | ∆*motA::kan*, *amyE::*P*_hag_-hag*^T209C^-*spec* | DS1895 was transformed with BKK13690 genomic DNA. |
| SH411 | ∆*CORE (fliO-flhA)::tet*, P*_fla/che_-flhF*, *amyE::*P*_hag_-hag*^T209C^-*spec* | DS1895 was transformed with SH9 genomic DNA. |
| SH415 | ∆*sigD::tet*, *amyE::*P*_hag_-hag*^T209C^-*spec* | DS1895 was transformed with IB97 genomic DNA. |
| SH418 | ∆*fliD::erm,* P*_yvyC-_fliS* | Constructed by Gibson master mix, to assemble PCR products, amplified from PY79 genomic DNA, using primers 5250-5251, 5252-5253, 5254-5255, and from pWX467 using primers 2430-2431. The mixture was transformed into PY79. |
| SH419 | ∆*motA::erm*, P*_motA-_motB* | Constructed by Gibson master mix, to assemble PCR products, amplified from PY79 genomic DNA, using primers 5244-5245, 5246-5247, 5248-5249, and from pWX467 using primers 2430-2431. The mixture was transformed into PY79. |
| SH422 | ∆*fliD::erm,* P*_yvyC-_fliS*/pLS20_cm_ | pLS20_cm_ was introduced into SH418 by conjugation with SH337. |
| SH423 | ∆*motA::erm*, P*_motA_-motB*/pLS20_cm_ | pLS20_cm_ was introduced into SH419 by conjugation with SH337. |
| SH436 | *amyE::*P*_hyper-spank_*-*rapA*_pLS20_-*spec* | PY79 was transformed with pSH55. |
| SH437 | *sacA::*P_C_-*gfp-spec* | Constructed by Gibson master mix, to assemble PCR products, amplified from PY79 genomic DNA, using primers 3334-3335, 3336-3337, and from AR16 genomic DNA, using primers 5418-3416, and from MBS7 DNA, using primers 5416-5417, and from pWX466 using primers 2430-2431. The mixture was transformed into PY79. |
| SH442 | ∆*ymdB::tet*/pLS20_cm_ | pLS20_cm_ was introduced into GB61 by conjugation with SH337. |
| SH443 | ∆*hag::erm*/pLS20_cm_ | pLS20_cm_ was introduced into GD215 by conjugation with SH337. |
| SH444 | *sacA::*P_C_-*gfp-spec*/pLS20_cm_ | SH337 was transformed with SH437 genomic DNA. |
| SH447 | ∆*ymdB*::*tet*, *sacA::*P_C_-*gfp-spec* /pLS20_cm_ | SH442 was transformed with SH437 genomic DNA. |
| SH448 | ∆*hag::erm*, *sacA::*P_C_-*gfp-spec*/pLS20_cm_ | SH443 was transformed with SH437 genomic DNA. |
| SH450 | *amyE::*P*_hyper-spank_*-*rapA*_pLS20_-*spec*/pLS20_cm_ | SH337 was transformed with SH436 genomic DNA. |
| SH451 | ∆*CORE (fliO-flhA)::tet*, P*_fla/che_-flhF*, *amyE::*P*_hyper-spank_-rapA_pLS20_-spec*/pLS20_cm_ | SH352 was transformed with SH436 genomic DNA. |
| SH452 | ∆*sigD::tet, amyE::*P*_hyper-spank_-rapA_pLS20_-spec*/pLS20_cm_ | PK143 was transformed with SH436 genomic DNA. |
| SH453 | ∆*hag::erm*, *amyE::P_hyper-spank_-rapA_pLS20_-spec*/pLS20_cm_ | SH443 was transformed with SH436 genomic DNA. |
| SH457 | ∆*ymdB::tet*, *sacA::*P_C_-*gfp-spec* | GB61 was transformed with SH437 genomic DNA. |
| SH458 | ∆*hag::erm*, *sacA::*P_C_-*gfp-spec* | GD215 was transformed with SH437 genomic DNA. |
| SH461 | WT/pLS20_cm_-*tie*_2xHA_-*erm* | Constructed by Gibson master mix, to assemble PCR products, amplified from MBS7 DNA, using primers 5557-5559, 5560-5561, and from SH347 DNA, using primers 5562-2431. The mixture was transformed into SH337. |
| SH464 | WT/pLS20_cm_-∆*tie*-*erm* | Constructed by Gibson master mix, to assemble PCR products, amplified from MBS7 DNA, using primers 5630-5631, 5560-5561, and from pWX467 using primers 2430-2431. The mixture was transformed into SH337. |
| SH481 | ∆*motA::kan*/pLS20_cm_-*tie*_2xHA_-*erm* | SH461 was transformed with BKK13690 genomic DNA. |
| SH483 | WT/pLS20_cm_-∆*tie* | SH464 was transformed with pDR244 to remove the *erm* gene. |
| SH484 | *sacA::*P_C_-*gfp-kan* | Constructed by Gibson master mix, to assemble PCR products, amplified from SH437 genomic DNA, using primers 3334-3416, and from SH345 genomic DNA, using primers 2430-3337. The mixture was transformed into PY79. |
| SH485 | *amyE::P_hyper-spank_-tie_pLS20_-spec*/pLS20_cm_-∆*tie* | SH483 was transformed with pSH58. |
| SH494 | *sacA::*P_33_-*gfp-kan* | Constructed by Gibson master mix, to assemble PCR products, amplified from PY79 genomic DNA, using primers 3334-3335, and from SH484 genomic DNA, using primers 5418-3337, and from MBS7 DNA, using primers 6261-6262. The mixture was transformed into PY79. |
| SH495 | ∆*flgM::kan* | PY79 was transformed with BKK35430 genomic DNA. |
| SH496 | ∆*flgM::kan/*pLS20_cm_ | pLS20_cm_ was introduced into SH495 by conjugation with SH337. |
| SH501 | *sacA::*P_33_-*gfp-kan*/pLS20_cm_ | pLS20_cm_ was introduced into SH494 by conjugation with SH337. |
| SH504 | ∆*ymdB::tet*, *sacA::*P_33_-*gfp-kan* | SH494 was transformed with GB61 genomic DNA. |
| SH505 | ∆*hag::erm*, *sacA::*P_33_-*gfp-kan* | SH494 was transformed with GD215 genomic DNA. |
| SH509 | ∆*ymdB::tet*, *sacA::*P_33_-*gfp-kan*/pLS20_cm_ | pLS20_cm_ was introduced into SH504 by conjugation with SH337. |
| SH510 | ∆*hag::erm*, *sacA::*P_33_-*gfp-kan*/pLS20_cm_ | pLS20_cm_ was introduced into SH505 by conjugation with SH337. |
| SH561 | ∆*flgM::kan*/pLS20_cm_-*ssb-yfp-erm* | pLS20_cm_-*ssb-yfp-erm* was introduced into SH495 by conjugation with SH347. |
| SH568 | ∆*hag::erm*, *sacA::*P*_veg_-mCherry*-*phleo* | BDR2637 was transformed with GD215 genomic DNA. |
| SH579 | *sacA*:*:*P_33_-*gfp-kan*/pLS20_cm_-*ssb-yfp-erm* | pLS20_cm_-*ssb-yfp-erm* was introduced into SH494 by conjugation with SH347. |
| SH582 | *amyE::*P*_hyper-spank_*-*epsE*-*spec*/pLS20_cm_-*tie_-_*_2xHA-_*erm* | SH461 was transformed with pLZ3. |
| SH590 | ∆*degU::kan* | PY79 was transformed with BKK35490 genomic DNA. |
| SH592 | ∆*degU::kan*/pLS20_cm_ | SH337 was transformed with SH590 genomic DNA. |
| SH593 | ∆*hag::erm*, ∆*degU::kan*/pLS20_cm_ | SH443 was transformed with SH590 genomic DNA. |
| SH609 | ∆*hag::erm*, ∆*degU::kan* | GD215 was transformed with SH590 genomic DNA. |
| SH637 | *amyE::*P*_rrnE_*-*gfp-spec*/pLS20_cm_-*tie*_2xHA_-*erm* | SH461 was transformed with AR16 genomic DNA. |
| OS2 | *B. megaterium* | (Stempler *et al*, 2017) |
| OS4 | *B. cereus* | Laboratory stock |
