# Supplementary material for: Flagellar rotation facilitates the transfer of a bacterial conjugative plasmid
Source: EMBO J. 2024 Dec 2;44(2):587–611. doi: 10.1038/s44318-024-00320-0 (PMC11730352; doi:10.1038/s44318-024-00320-0)
Supplement: Supplementary file 2 — Table EV2 [file 44318_2024_320_MOESM2_ESM.docx]

**Table EV2: List of primers used in this study**

| **Primer name** | **Primer sequence (5'-3')** |
| --- | --- |
| 2430 | TTCTGCTCCCTCGCTCAG |
| 2431 | CAGGGAGCACTGGTCAAC |
| 2513 | CACAGCTTGATGTGCAGATTACGCCG |
| 2514 | CTGAGCGAGGGAGCAGAATGTTTTGTTCCTCCCTGAATATGTTG |
| 2516 | GTTGACCAGTGCTCCCTGTAATTTTAAAAAAGACCTTGGCGTTGCCAG |
| 2517 | CAACAACGTTAAATGCCAAATTGTAATC |
| 3334 | CAGCATTTTCCGCTTTTCTC |
| 3335 | AGCCTGCCCTTTCAAATTCT |
| 3336 | GTTGACCAGTGCTCCCTGTGTTTTCCCTTCAAGGATCG |
| 3337 | TTTTCCAAACATTCCGGTGT |
| 3416 | CTGAGCGAGGGAGCAGAATTATTTGTATAGTTCATCCATGCCA |
| 5122 | TTGGAGGCACTTCTGAAAGC |
| 5123 | CTGAGCGAGGGAGCAGAATGTTCACGATCCTTTTCTTTTTACT |
| 5124 | GTTGACCAGTGCTCCCTGTTTTATCGCGGAAAATAAACG |
| 5125 | GCTGCATTATCTGCGAATTTT |
| 5126 | GGAAAAATTGGTTTAACAACGA |
| 5127 | CTGAGCGAGGGAGCAGAAGATTCTCCTCCAATCGGATG |
| 5128 | GTTGACCAGTGCTCCCTGAGTGAGGATTTTTTTATTTTTGTATTAACA |
| 5129 | CACGGATACGAGACTCAGCA |
| 5130 | GTTGACCAGTGCTCCCTGAAAGACCTTGGCGTTGCC |
| 5131 | CACTGTTTTTGCTGTTGTTTCG |
| 5141 | CGGAGCACAACGAAATGTTA |
| 5142 | GGAGGCTTACTTGTCTGCTTTC |
| 5143 | ATGAGATAATGCCGACTGTACTTTT |
| 5144 | CCCGTTTTAATGCAGTTCCA |
| 5145 | GAAAGCAGACAAGTAAGCCTCCATGCAAGGGTTTATTGTTTTCTAA |
| 5146 | AAAAGTACAGTCGGCATTATCTCATTTATTTGTATAGTTCATCCATGCCA |
| 5171 | AGTTCTTCTCCTTTACTCATAAAAGGAAGGTCATCATCACTAA |
| 5172 | ATGAGTAAAGGAGAAGAACTTTTCACT |
| 5174 | CCCTTATGTACCAAGGGGT |
| 5175 | GTTGACCAGTGCTCCCTGTGACACGAAAGGAGAATTTAGACC |
| 5176 | TTTCTGTTTCGGATCGGTTC |
| 5179 | CTGAGCGAGGGAGCAGAAAGCCTGCCCTTTCAAATTCT |
| 5244 | CCATGAGGAAGCTGGACAAT |
| 5245 | CTGAGCGAGGGAGCAGAAAGTTTTCACCAAATCCTTTTTTAC |
| 5246 | ATGGCGAGAAAAAAGAAGAAGAAG |
| 5247 | GATCACGCTGCTGAAATTGA |
| 5248 | GTTGACCAGTGCTCCCTGTGCAAGCAAGCTGCTAATTT |
| 5249 | CTTCTTCTTCTTTTTTCTCGCCATCATAGTTTTCACCAAATCCTTTTT |
| 5250 | TTGAGGATATGGGTGCTGAC |
| 5251 | CTGAGCGAGGGAGCAGAACTCAAACCACTCCTATTCTACTTTTT |
| 5252 | ATGTAATTTGGAGGATGACACATG |
| 5253 | TCACACAAATTTCAGCACACC |
| 5254 | GTTGACCAGTGCTCCCTGTCTCAAGCTATGCTTGCTCA |
| 5255 | CATGTGTCATCCTCCAAATTACATTTGATCATCCCCTATGCCC |
| 5416 | AGAATTTGAAAGGGCAGGCTTTATACCACCTCGCAAAATAAACCCTG |
| 5417 | CCAGTGAAAAGTTCTTCTCCTTTACTCATCAAGCTATTCCCTCCCTAAATTTTCAATC |
| 5418 | ATGAGTAAAGGAGAAGAACTTTTCACTGG |
| 5419 | TTGTGAGCGGATAACAATTAAAAGGTGGTGAACTACTATGTTGTCCAAAGTAAAAAAAGTACCGTCTC |
| 5420 | GTTTCCACCGAATTAGCTTGCATGTCATCCTAACGCCTCCGTTATTC |
| 5557 | TCCGGCTGCTGATGGTCTGCC |
| 5559 | CATAGGGATAGCCAGCGTAATCTGGAACATCATATGGGTATTTCTTCAACCAATTAATATCACTTTGTGACCAGCCG |
| 5560 | GTTGACCAGTGCTCCCTGCCTTTTCAACTGTTTGGAGGAAAAAGTATGGAG |
| 5561 | TCCGCCGTCTGCTGTCTTTAAAAAATC |
| 5562 | TTACGCTGGCTATCCCTATGACGTCCCGGACTACGCATAATTCTGCTCCCTCGCTCAG |
| 5630 | CTAAGTCTGCGACTGGGCAATATACG |
| 5631 | CTGAGCGAGGGAGCAGAATTCGATCACCCCTTTCTTATTGTGCTG |
| 5642 | TTGTGAGCGGATAACAATTAAAAGGTGGTGAACTACTATGGTGATTCACATAGTGTATAAAAGAATCTTG |
| 5643 | GTTTCCACCGAATTAGCTTGCATGTTATTTCTTCAACCAATTAATATCACTTTGTGACCAGC |
| 6261 | AGAATTTGAAAGGGCAGGCTCTATACATCGGACTTTATATTCTACTTG |
| 6262 | CCAGTGAAAAGTTCTTCTCCTTTACTCATCATTTTTCCACCTCATTTTCC |
| 7054 | GTTAAAAAAATGTCTGTTATCTTAGTGAAAGAGTCAGTGA |
| 7055 | CATAGGGATAGCCAGCGTAATCTGGAACATCATATGGGTATTCAGAAATTACTTTTTGTAGCAAATAGTTAGGATTAGGA |
| 7056 | GTTGACCAGTGCTCCCTGAGTGAGGTTGATCAGGATTCACACG |
| 7057 | GACTCTTGATCTTCAATTCTCTTTTTTAGCGTTTCAAG |
| 7478 | TTGTGAGCGGATAACAATTAAAAGGTGGTGAACTACTATGAACTCAGGACCGAAAGT |
| 7479 | GTTTCCACCGAATTAGCTTGCATGCTATTCATGCTTGACAAGCC |
| Primers used for qRT-PCR | |
| 5915  (*ses* F) | TATTGTTGCCGGTGCAATTA |
| 5916  (*ses* R) | TTCTTTGAAACCCCATCAGC |
| 5917  (*tie* F) | CCATTAAGCTGAACGGGAAA |
| 5918  (*tie* R) | CCAGCTGTCTGATCTCACCA |
| 6197 (*conAn1* F) | GTTTGACGGCAGCACTCATG |
| 6198 (*conAn1* R) | GGGTTACTTTTCTTGCCGGC |
| 6199  (*gene 30* F) | TGCACAGATTGAGGAAAGGCA |
| 6200  (*gene 30* R) | TCCGTTCTTCAGTCATATGTGCA |
| 6201  (*gene 31* F) | AGTGAGAGCTGGGATCGCA |
| 6202  (*gene 31* R) | TAACCACGCGCTTACCATCA |
| 6203  (*gene 32* F) | CCGCCGACAATTGAAGTACC |
| 6204  (*gene 32* R) | CTTCCCGCCATGCCAATTTC |
| 6205  (*gene 33* F) | AGAGGGACGCCTAGAATTCAA |
| 6206  (*gene 33* R) | CCTTTAATTCACCATTCTTCTCGGT |
| 6207  (*virB11* F) | GTTGCAGCAAAAGCTAACATTGG |
| 6208  (*virB11* R) | CCCAGGAGAAGTAAGCCAGC |
| 6209  (*virD4* F) | GAAGTCGCTAATATCCCGCCA |
| 6210  (*virD4* R) | TCCTCCCACTCGTCACTCAT |
| 6249  (*rapA* F) | TTCGTGATGCCTTGACTGAG |
| 6250  (*rapA* R) | TTGCTGTCCTTTAGGCGAAT |
