# Supplementary material for: Flagellar rotation facilitates the transfer of a bacterial conjugative plasmid
Source: EMBO J. 2024 Dec 2;44(2):587–611. doi: 10.1038/s44318-024-00320-0 (PMC11730352; doi:10.1038/s44318-024-00320-0)
Supplement: Supplementary file 3 — Appendix [file 44318_2024_320_MOESM3_ESM.pdf]

APPENDIX

Flagellar rotation facilitates the transfer of a bacterial conjugative plasmid

Saurabh Bhattacharya[1,2], Michal Bejerano-Sagie[1,2], Miriam Ravins[1], Liat Zeroni[1], Prabhjot Kaur[1], Venkadesaperumal Gopu[1], Ilan Rosenshine[1,3] and Sigal Ben-Yehuda[1,3]

[1]Department of Microbiology and Molecular Genetics

Institute for Medical Research Israel-Canada (IMRIC)

The Hebrew University-Hadassah Medical School, POB 12272

The Hebrew University of Jerusalem, 91120

Jerusalem, Israel.

[2] These authors contributed equally to this work.

[3] Co-correspondence: sigalb@ekmd.huji.ac.il (S. B-Y); ilanr@ekmd.huji.ac.il (I. R)

TABLE OF CONTENTS

Appendix Figure S1: Developing a system for visualizing pLS20 conjugation events

Appendix Figure S2: MCs are pLS20 and flagella dependent

Appendix Figure S3: Exploring the mechanism of flagella-mediated conjugation

Appendix Figure S1

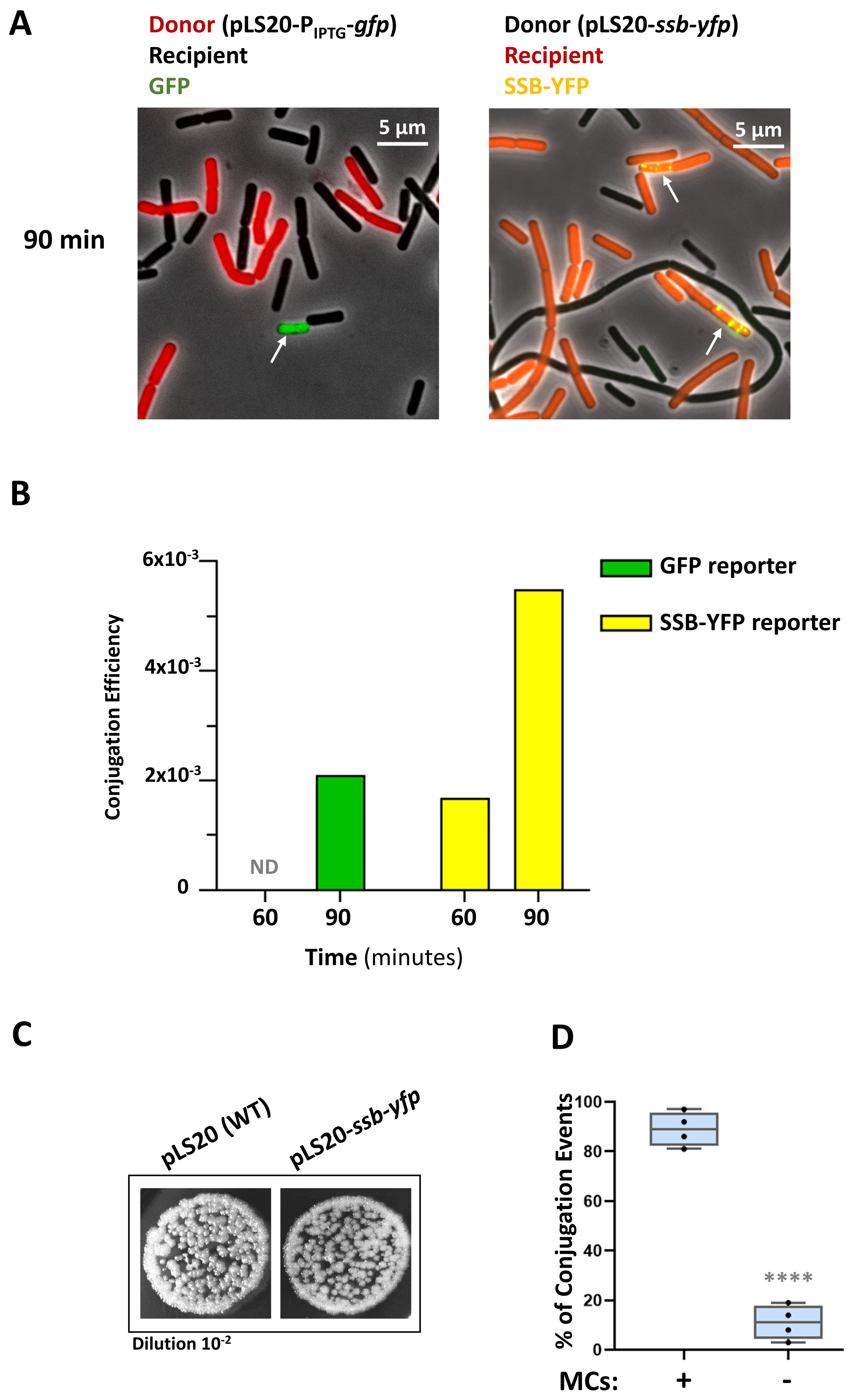

Appendix Figure S1: Developing a system for visualizing pLS20 conjugation events

A. Left panel: Donor cells (red) (SH363: WT, *sacA::*P_{veg}*-mCherry, amyE::lacI*/$pLS20_{spec}$-P_{IPTG}-*gfp*) were mixed with recipient cells (dark) (PY79:WT), incubated for 90 minutes and visualized by fluorescence microscopy. Shown is an overlay image of phase contrast (grey) with fluorescence from mCherry (red) and GFP (green). GFP synthesis from $pLS20_{spec}$-P_{IPTG}-*gfp* is repressed in donor bacteria by LacI that is lacking from recipient cells. Thus, recipient cells, expressing GFP, were scored as transconjugants. Right panel: Donor cells (dark) (SH347: WT/$pLS20_{cm}$-*ssb-yfp*) were mixed with recipient cells (red) (BDR2637: *sacA::*P_{veg}*-mCherry*) in 1:1 ratio, incubated for 90 minutes and visualized by fluorescence microscopy. Shown is an overlay image of phase contrast (grey), with fluorescence from mCherry (red) and SSB-YFP (yellow). Recipient cells, expressing mCherry and displaying SSB-YFP foci, were scored as transconjugants. Arrows highlight transconjugant cells. Representative images out of 3 independent biological repeats.

B. Conjugation efficiencies of the strains described in (A) were calculated as the number of transconjugants (T)/number of total recipients (T+R) at the indicated time points. Plotted are conjugation efficiencies derived from a representative experiment out of 3 independent biological repeats. n>1000 cells for each strain. ND-not detected.

C. Donor strains: (SH337: WT/$pLS20_{cm}$) and (SH347: WT/$pLS20_{cm}$-*ssb-yfp*) were mixed with recipient cells (SH345: *sacA::kan*) in 1:1 ratio, and incubated for 20 minutes. Shown are images of spotted conjugation mixtures (10^{-2} dilution) over LB agar containing chloramphenicol and kanamycin, selecting for transconjugants. Representative images out of 3 independent biological repeats.

D. Donor cells (SH347: WT/$pLS20_{cm}$-*ssb-yfp*) were mixed with recipient cells (BDR2637: *sacA::*P_{veg}-*mCherry*) in 1:1 ratio, placed over semi-solid (0.6%) LB agarose pads, incubated for 80 minutes, and visualized by fluorescence microscopy. Conjugation events represented by transconjugant

cells expressing SSB-YFP were quantified and categorized based on their association with MCs. At least 4 independent biological repeats were conducted. Data are shown as box plot graphs. The box is determined by the 25th and 75th percentiles, and whiskers are determined by min and max; the line in the box indicates the median. Statistical significance was calculated using paired t-tests. P-value: (****) ≤ 0.0001.

Appendix Figure S2

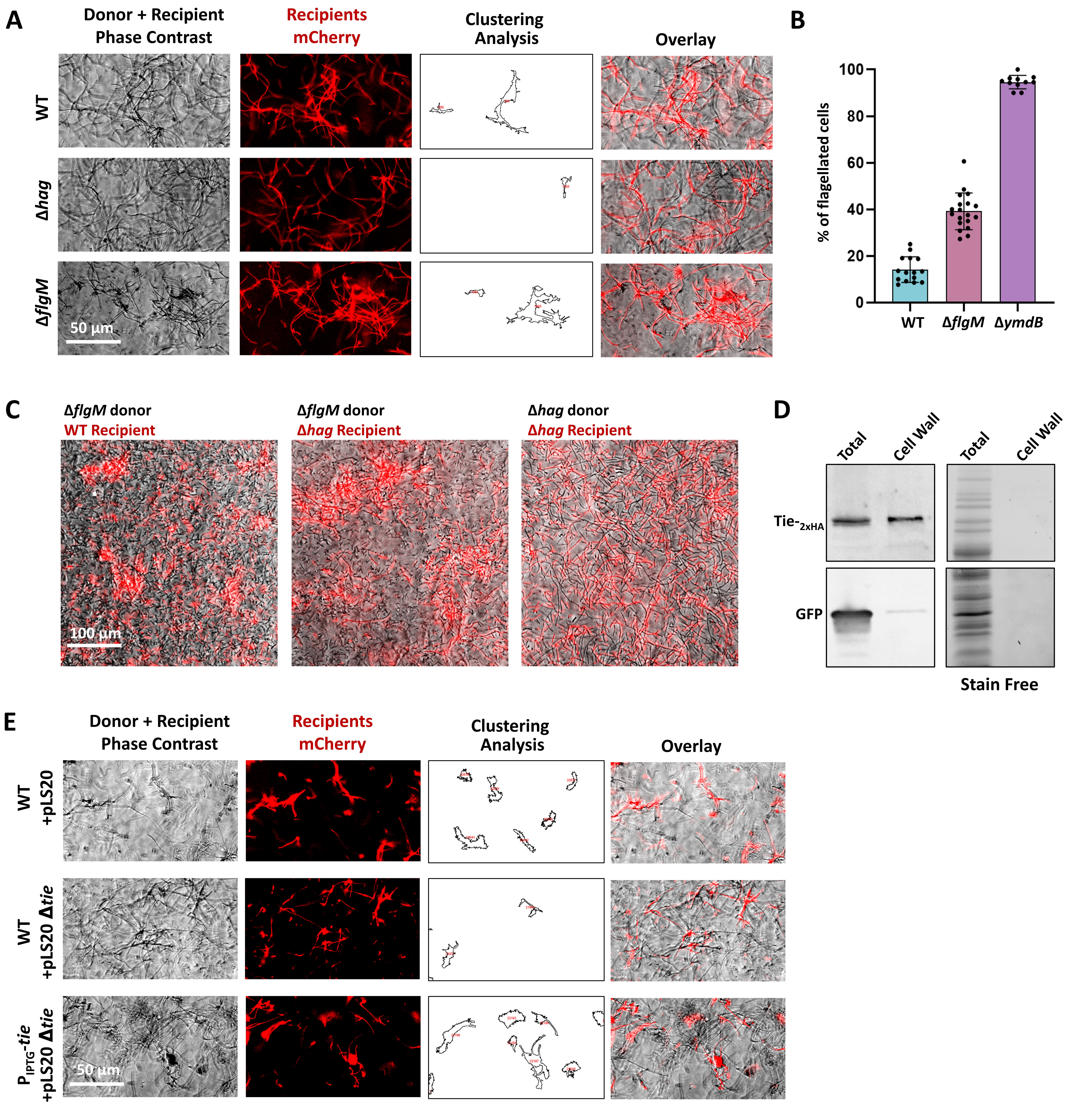

Appendix Figure S2: MCs are pLS20 and flagella dependent

A. Donor strains (dark) WT (SH337), Δ*hag* (SH443), or Δ*flgM* (SH496) harboring pLS20$_{cm}$, were mixed with recipient cells (red) (BDR2637: *sacA::*P$_{veg}$-*mCherry*) in 1:1 ratio, and the formation of MCs in liquid medium was followed using digital wide-field microscopy. Shown are representative images captured at time point 30 minutes after mixing: phase contrast (grey), fluorescence from mCherry-labeled recipients (red), computed clustering analysis derived from images of mCherry channel subjected to thresholding and analyzed for particles larger than 500 pixels^2 (outlines), and overlay of phase contrast and mCherry fluorescence. Quantification of this experiment is shown in Fig. 1F. A representative experiment out of 3 independent biological repeats.

B. The following strains: WT (DS1895), Δ*flgM* (SH408), and Δ*ymdB* (SH101), harboring modified flagellin *hag*^{T209C} (*amyE*::P$_{hag}$-*hag*^{T209C}), were grown in liquid LB, flagellin was stained with Alexa Fluor 594 C$_5$ maleimide, and cells were visualized by fluorescence microscopy (representative images are shown in Fig. 2C). Plotted are % of flagellated cells derived from a representative experiment out of 3 independent biological repeats. n>380 for each strain.

C. Donor strains (dark) Δ*flgM* (SH496) or Δ*hag* (SH443), harboring pLS20$_{cm}$, were mixed with recipient cells (red) WT (BDR2637: *sacA::*P$_{veg}$-*mCherry*) or Δ*hag* (SH568: Δ*hag, sacA::*P$_{veg}$-*mCherry*) in 1:1 ratio, and the formation of MCs in liquid medium was followed using digital wide-field microscopy. Shown are representative overlay images of phase contrast (grey) and fluorescence from mCherry-labeled recipients (red) captured 30 minutes after mixing. A representative experiment out of 3 independent biological repeats.

D. Whole cell lysates (Total) and cell wall proteins were extracted from donor cells (SH637: *amyE*::P$_{rrnE}$-*gfp/* pLS20$_{cm}$-*tie*-$_{2xHA}$) harboring pLS20$_{cm}$-tie$_{2xHA}$ and constitutively expressing cytoplasmic GFP. Samples were subjected to western blot analysis using anti-HA and anti-GFP antibodies (left panels). Stain-free total protein analysis is presented for comparison (right panels),

with only a faint signal detected from the cell wall fractions. Cytoplasmic GFP was hardly detected in the cell wall fraction despite its high abundance in the cell lysate (Total). Shown is a representative experiment out of 3 independent biological repeats.

E. Donor strains (dark): WT (SH337: pLS20$_{cm}$), Δ*tie* (SH483: pLS20$_{cm}$-Δ*tie*), and a *tie* complementing strain (SH485: *amyE*::P$_{IPTG}$-*tie*/pLS20$_{cm}$-Δ*tie*) were grown in the presence of IPTG (1 mM) and mixed with recipient cells (red) (BDR2637: *sacA::*P$_{veg}$-*mCherry*) in 1:1 ratio, and the formation of MCs in liquid medium was followed using digital wide-field microscopy. Shown are representative images captured at time point 30 minutes after mixing: phase contrast (grey), fluorescence from mCherry-labeled recipients (red), computed clustering analysis derived from images of mCherry channel subjected to thresholding and analyzed for particles larger than 500 pixels^2 (outlines), and overlay of phase contrast and mCherry fluorescence. Quantification of this experiment is shown in Fig. 5E. A representative experiment out of 3 independent biological repeats.

Appendix Figure S3

A

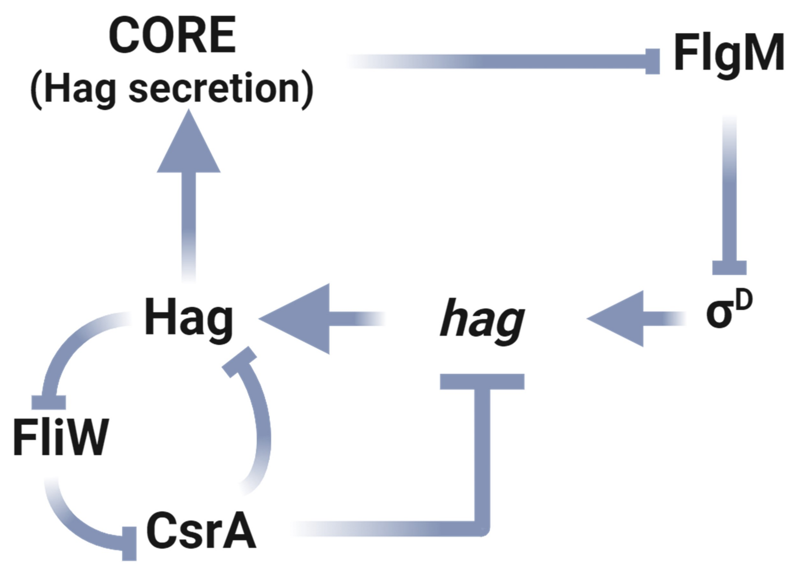

B

Donor genotype	Conjugation (% of WT)	Dilution 10^{-2}
WT	100	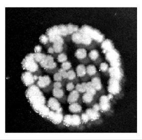
ΔfliW	95	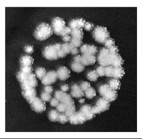
ΔcsrA	70	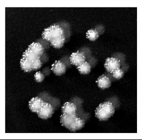
ΔfliW ΔcsrA Δhag	1	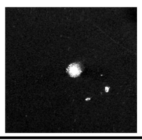
ΔcsrA Δhag	4	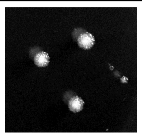
ΔcsrA ΔflgM	156	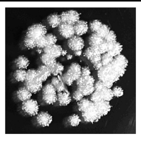
*ΔfliD**	1	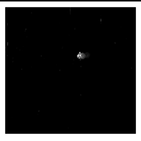

C

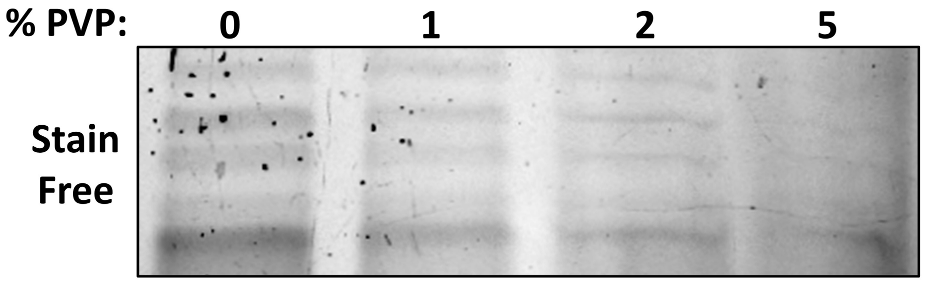

D

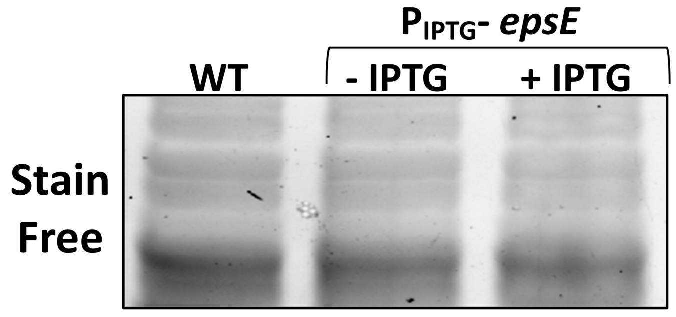

Appendix Figure S3: Exploring the mechanism of flagella-mediated conjugation

A. Schematics depicting the regulatory circuits in *B. subtilis* cells governing flagella biosynthesis via molecular regulation of *hag* at the transcriptional and translational levels. Arrows highlight activation and T-bars indicate inhibition. Adapted from (Oshiro *et al*, 2019).

B. Donor strains: WT (SH337), Δ*fliW* (GV289), Δ*csrA* (GV290), Δ*fliW* Δ*csrA* Δ*hag* (GV291), Δ*csrA* Δ*hag* (GV293), Δ*csrA* Δ*flgM* (SH381), and Δ*fliD* (SH422), harboring $pLS20_{cm}$ were mixed with recipient cells (SH345: *sacA::kan*) in 1:1 ratio, incubated for 20 minutes, and serial dilutions were spotted either on LB agar containing chloramphenicol and kanamycin or solely kanamycin, selecting for transconjugants and recipients, respectively. Shown are images of spotted conjugation mixtures (10^{-2} dilution) over LB agar containing chloramphenicol and kanamycin, selecting for transconjugants. Indicated conjugation efficiencies were calculated as % of WT conjugation efficiency. A representative experiment out of 3 independent biological repeats.

**fliD* encodes a homolog of flagellar filament cap protein, serving as an extra-cytoplasmic chaperone for polymerization of Hag.

C. Whole cell lysates were extracted from WT donor cells (SH461: WT/$pLS20_{cm}$-*tie*$_{\text{-2xHA}}$), grown in LB medium supplemented with different concentrations of PVP. Samples were subjected to SDS-PAGE and stain-free total protein analysis. The corresponding western blot analysis using anti-HA antibodies is presented in Fig. 6D.

D. Whole cell lysates were extracted from donor strains WT (SH461: WT/ $pLS20_{cm}$-*tie*$_{\text{-2xHA}}$) and P_{IPTG}-*epsE* (SH582: *amyE*::P_{IPTG}-*epsE*/$pLS20_{cm}$-*tie*$_{\text{-2xHA}}$), grown in the absence or presence of IPTG (1 mM). Samples were subjected to SDS-PAGE and stain-free total protein analysis. The corresponding western blot analysis using anti-HA antibodies is presented in Fig. 6H.

References

Oshiro RT, Rajendren S, Hundley HA & Kearns DB (2019) Robust stoichiometry of FliW *subtilis*. *mBio* 10(3): e00533-19
