# Supplementary figures and images for: Flagellar rotation facilitates the transfer of a bacterial conjugative plasmid
Source: EMBO J. 2024 Dec 2;44(2):587–611. doi: 10.1038/s44318-024-00320-0 (PMC11730352; doi:10.1038/s44318-024-00320-0)

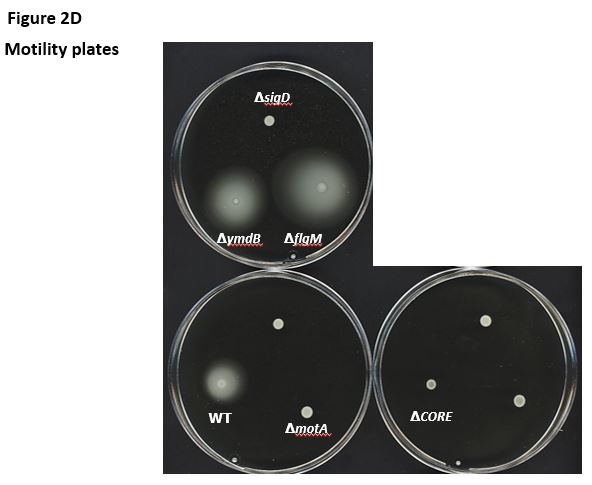

Supplement: Supplementary file 6 — Source data Fig. 2 [file 44318_2024_320_MOESM6_ESM.zip › Figure 2/2D/images of motility plates.JPG]

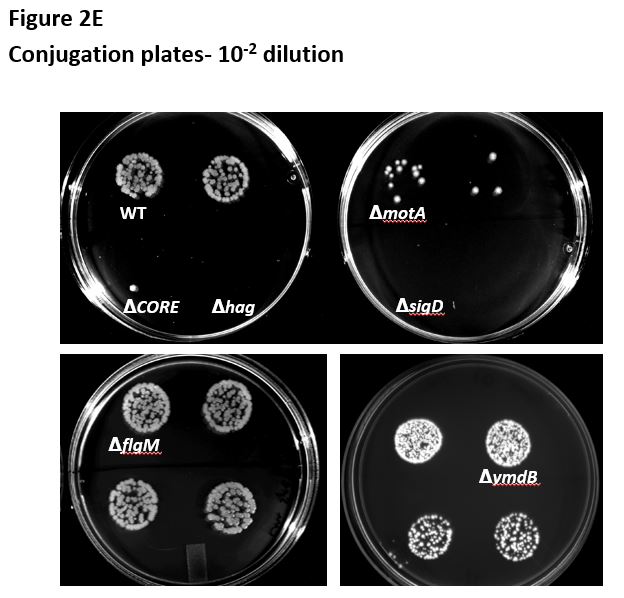

Supplement: Supplementary file 6 — Source data Fig. 2 [file 44318_2024_320_MOESM6_ESM.zip › Figure 2/2E/conjugation plates.JPG]

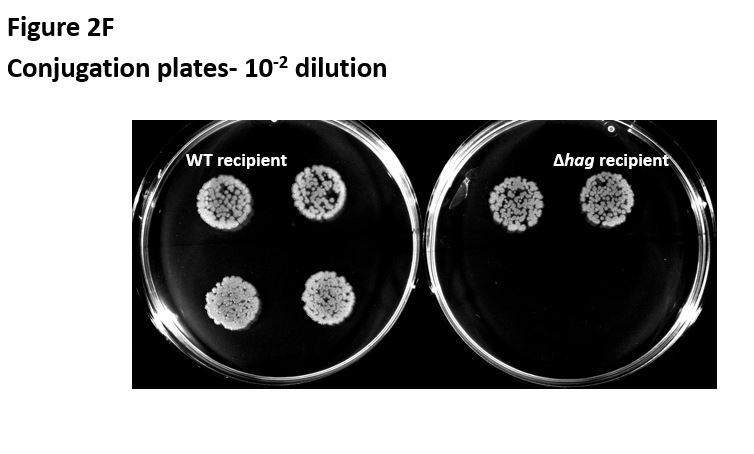

Supplement: Supplementary file 6 — Source data Fig. 2 [file 44318_2024_320_MOESM6_ESM.zip › Figure 2/2F/conjugation plates.JPG]

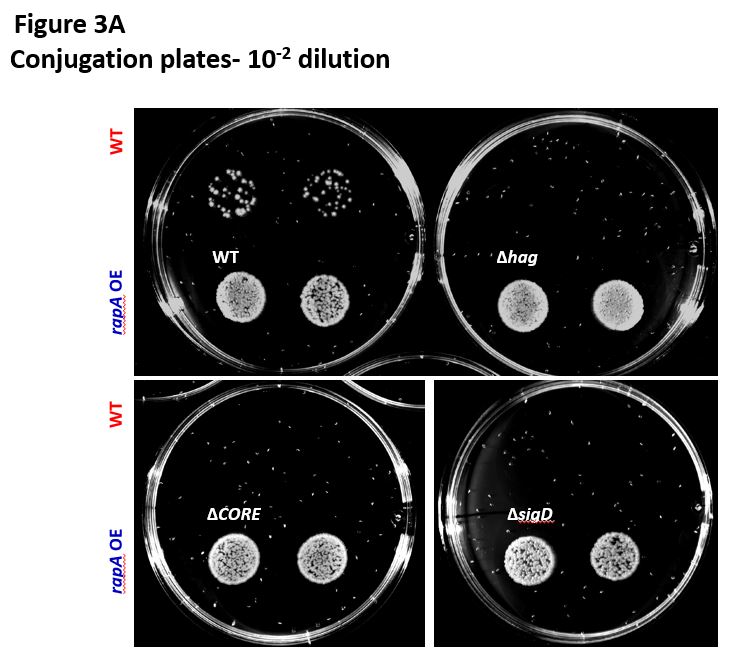

Supplement: Supplementary file 7 — Source data Fig. 3 [file 44318_2024_320_MOESM7_ESM.zip › Figure 3/3A/conjugation plates.JPG]

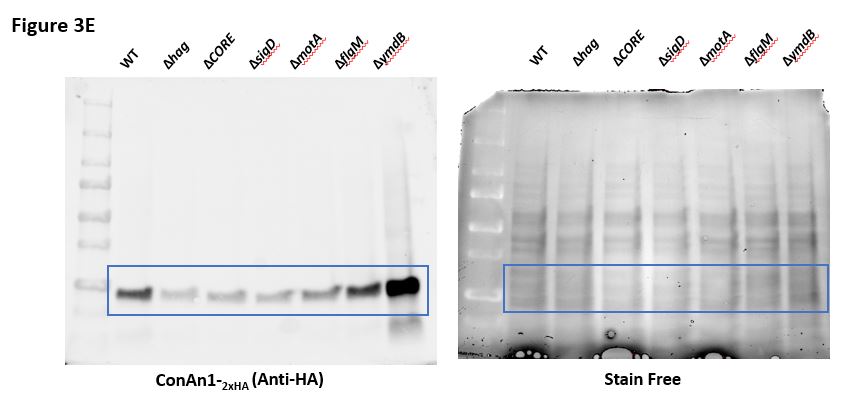

Supplement: Supplementary file 7 — Source data Fig. 3 [file 44318_2024_320_MOESM7_ESM.zip › Figure 3/3E/ConAn1 Western Blot.JPG]

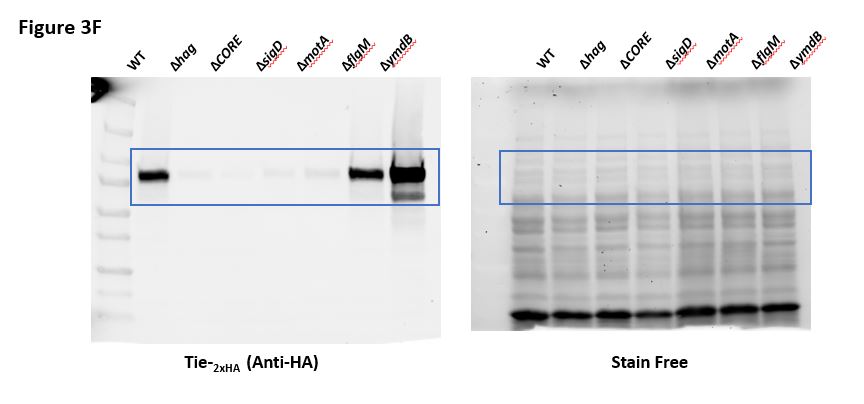

Supplement: Supplementary file 7 — Source data Fig. 3 [file 44318_2024_320_MOESM7_ESM.zip › Figure 3/3F/Tie Western Blot.JPG]

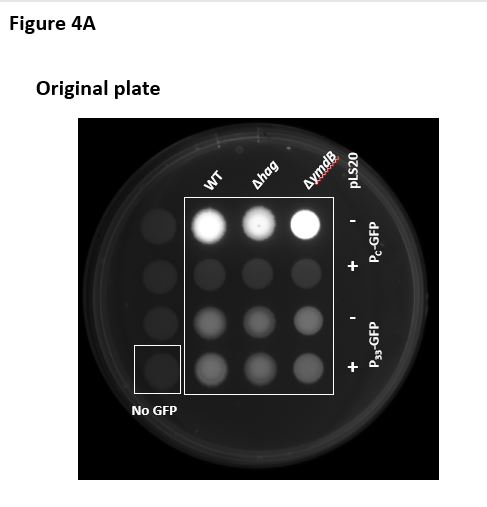

Supplement: Supplementary file 8 — Source data Fig. 4 [file 44318_2024_320_MOESM8_ESM.zip › Figure 4/4A/Plate flourescence Image.JPG]

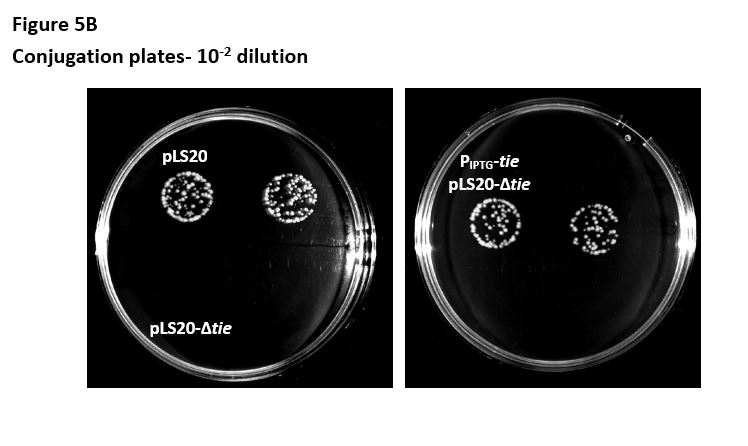

Supplement: Supplementary file 9 — Source data Fig. 5 [file 44318_2024_320_MOESM9_ESM.zip › Figure 5/5B/conjugation plates.JPG]

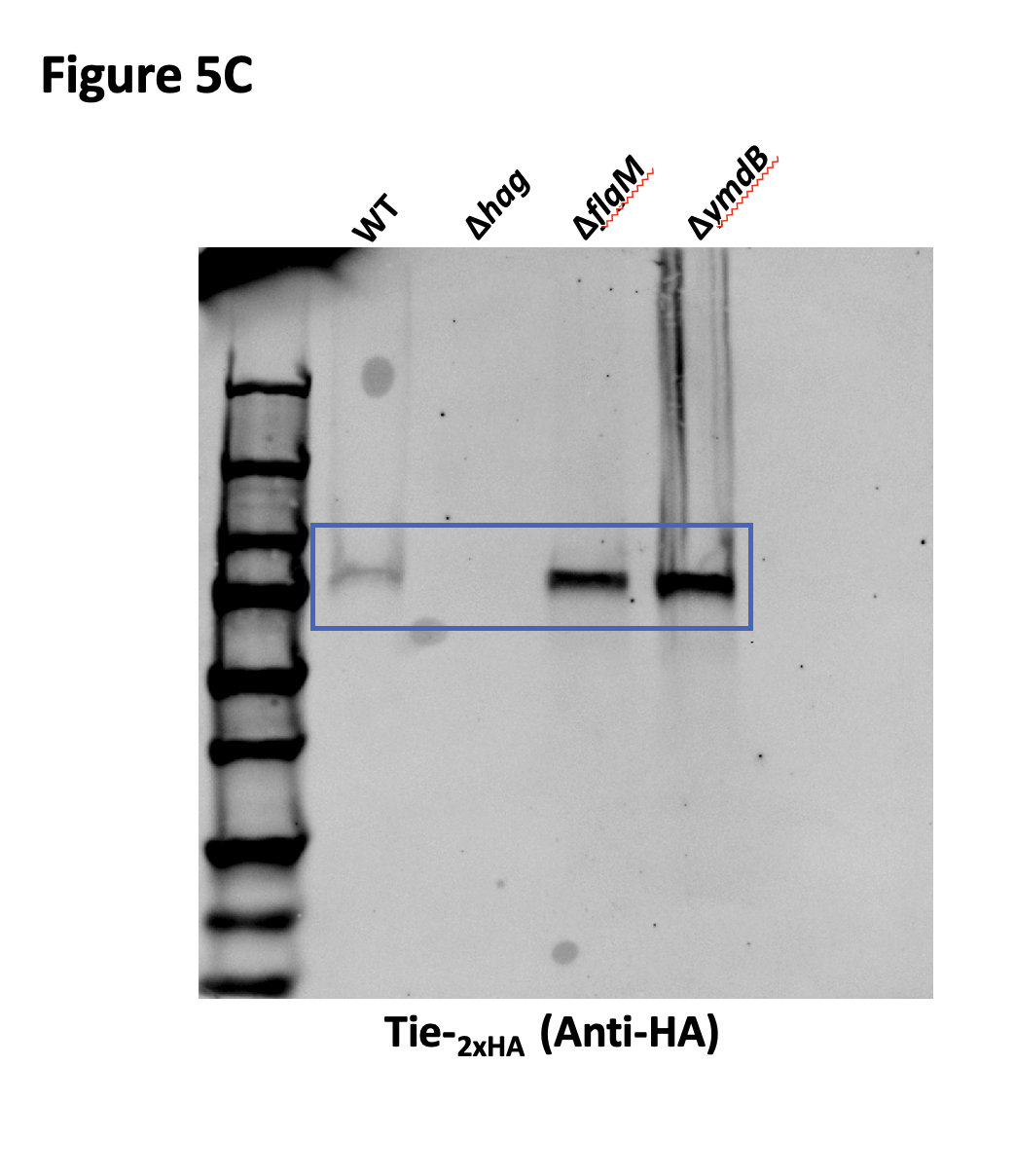

Supplement: Supplementary file 9 — Source data Fig. 5 [file 44318_2024_320_MOESM9_ESM.zip › Figure 5/5C/Cell wall Tie Western Blot.png]

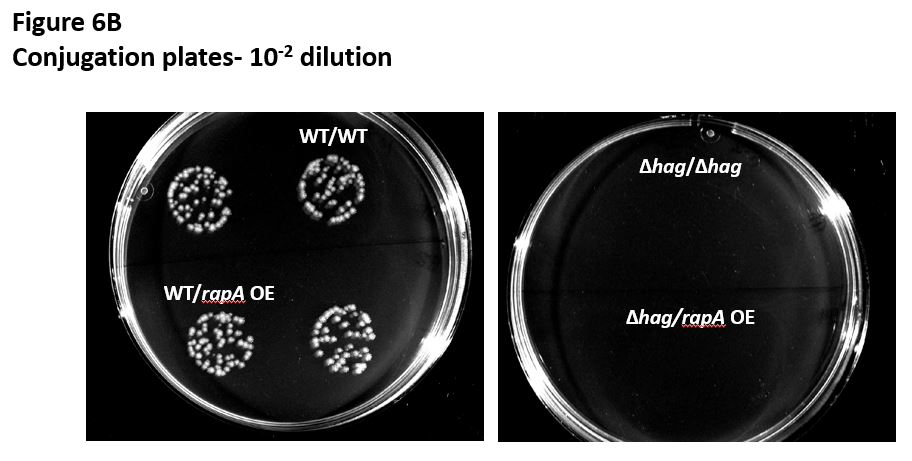

Supplement: Supplementary file 10 — Source data Fig. 6 [file 44318_2024_320_MOESM10_ESM.zip › Figure 6/6B/conjugation plates.JPG]

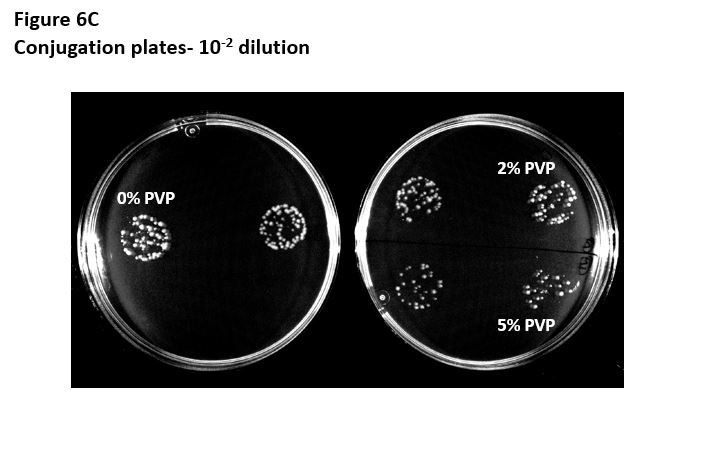

Supplement: Supplementary file 10 — Source data Fig. 6 [file 44318_2024_320_MOESM10_ESM.zip › Figure 6/6C/conjugation plates.JPG]

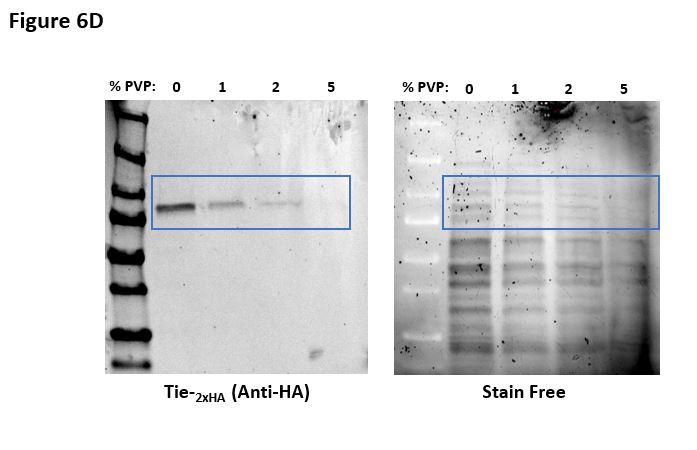

Supplement: Supplementary file 10 — Source data Fig. 6 [file 44318_2024_320_MOESM10_ESM.zip › Figure 6/6D/Tie Western Blot.JPG]

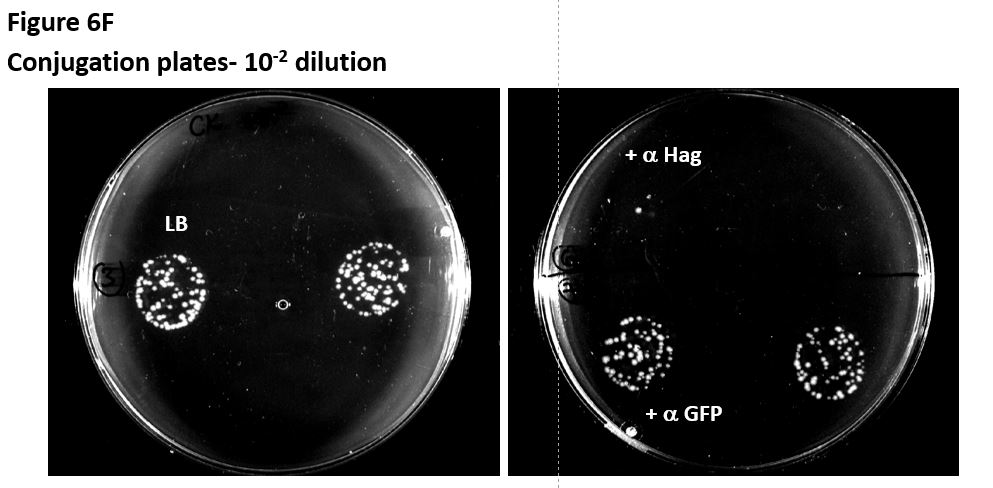

Supplement: Supplementary file 10 — Source data Fig. 6 [file 44318_2024_320_MOESM10_ESM.zip › Figure 6/6F/conjugation plates.JPG]

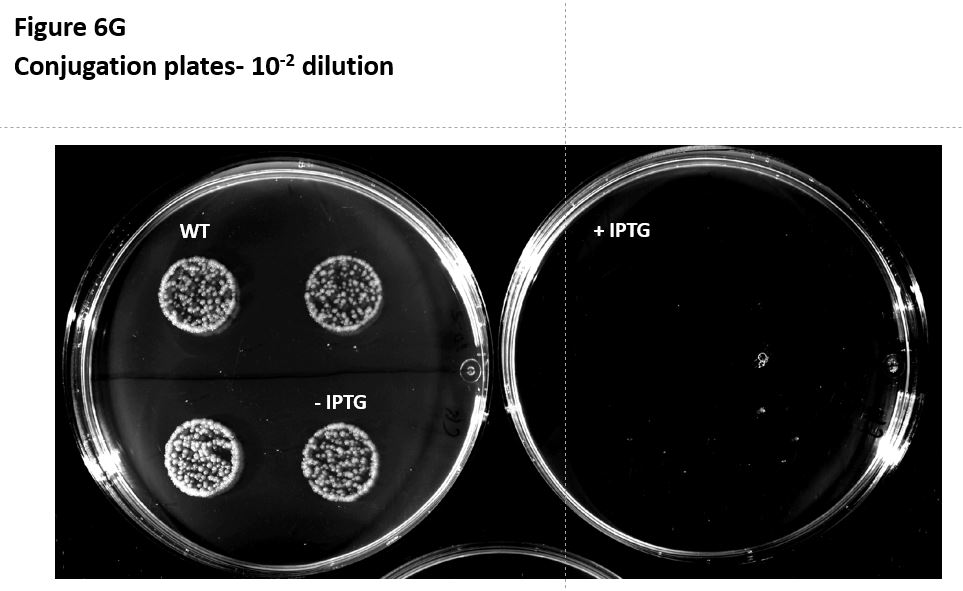

Supplement: Supplementary file 10 — Source data Fig. 6 [file 44318_2024_320_MOESM10_ESM.zip › Figure 6/6G/conjugation plates.JPG]

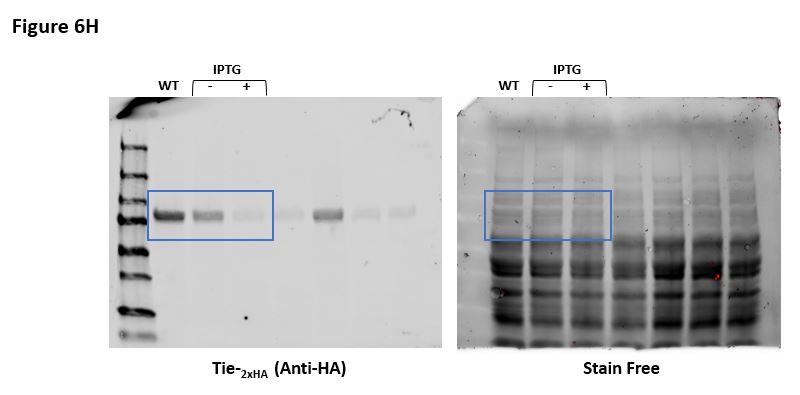

Supplement: Supplementary file 10 — Source data Fig. 6 [file 44318_2024_320_MOESM10_ESM.zip › Figure 6/6H/Tie Western Blot.JPG]

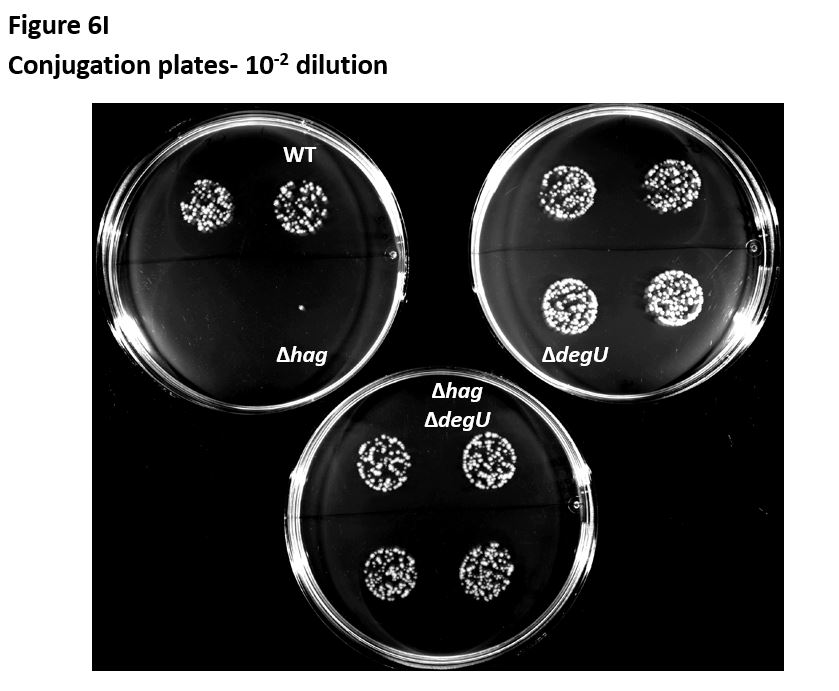

Supplement: Supplementary file 10 — Source data Fig. 6 [file 44318_2024_320_MOESM10_ESM.zip › Figure 6/6I/conjugation plates.JPG]

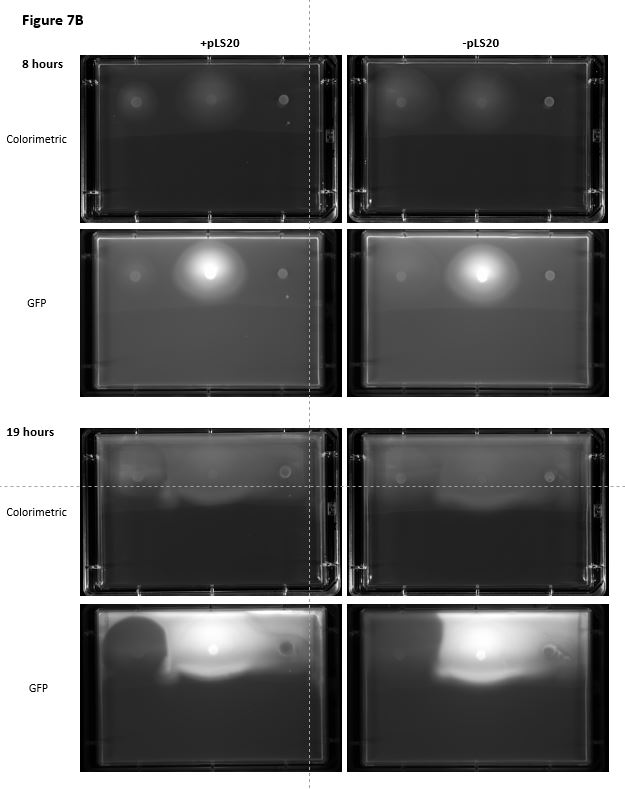

Supplement: Supplementary file 11 — Source data Fig. 7 [file 44318_2024_320_MOESM11_ESM.zip › Figure 7/7B/soft agar plates 8-19 hours.JPG]

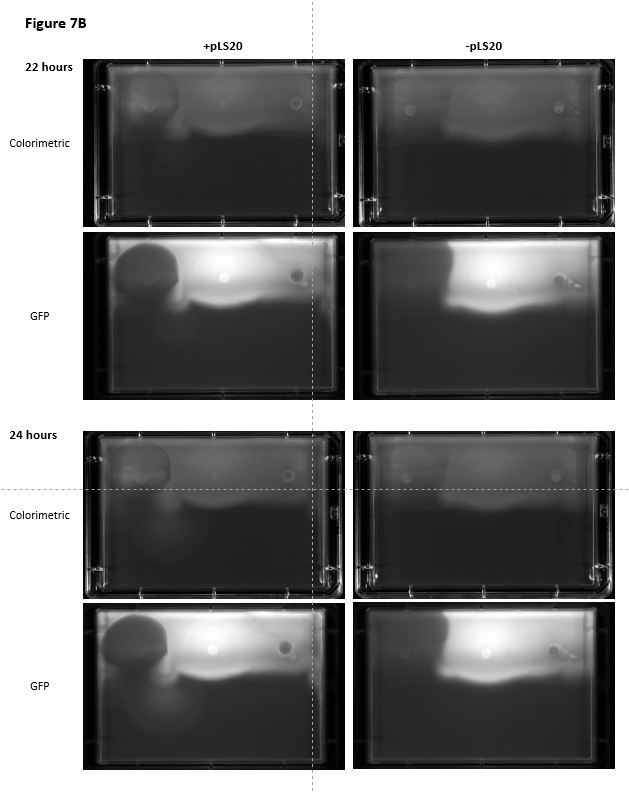

Supplement: Supplementary file 11 — Source data Fig. 7 [file 44318_2024_320_MOESM11_ESM.zip › Figure 7/7B/soft agar plates 22-24 hours.JPG]

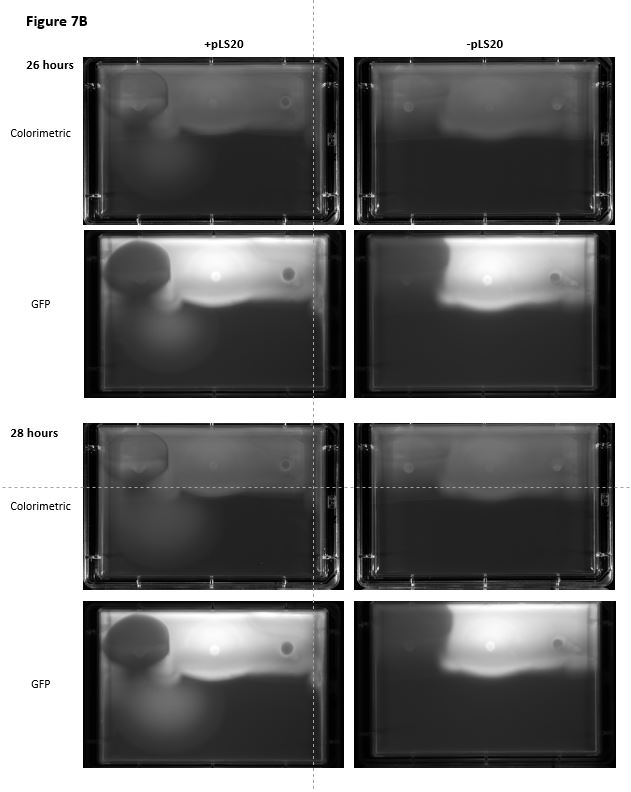

Supplement: Supplementary file 11 — Source data Fig. 7 [file 44318_2024_320_MOESM11_ESM.zip › Figure 7/7B/soft agar plates 26-28 hours.JPG]
