# Supplementary material for: Flagellar rotation facilitates the transfer of a bacterial conjugative plasmid
Source: EMBO J. 2024 Dec 2;44(2):587–611. doi: 10.1038/s44318-024-00320-0 (PMC11730352; doi:10.1038/s44318-024-00320-0)
Supplement: Supplementary file 12 — Expanded View Figures [file 44318_2024_320_MOESM12_ESM.pdf]

Expanded View Figures

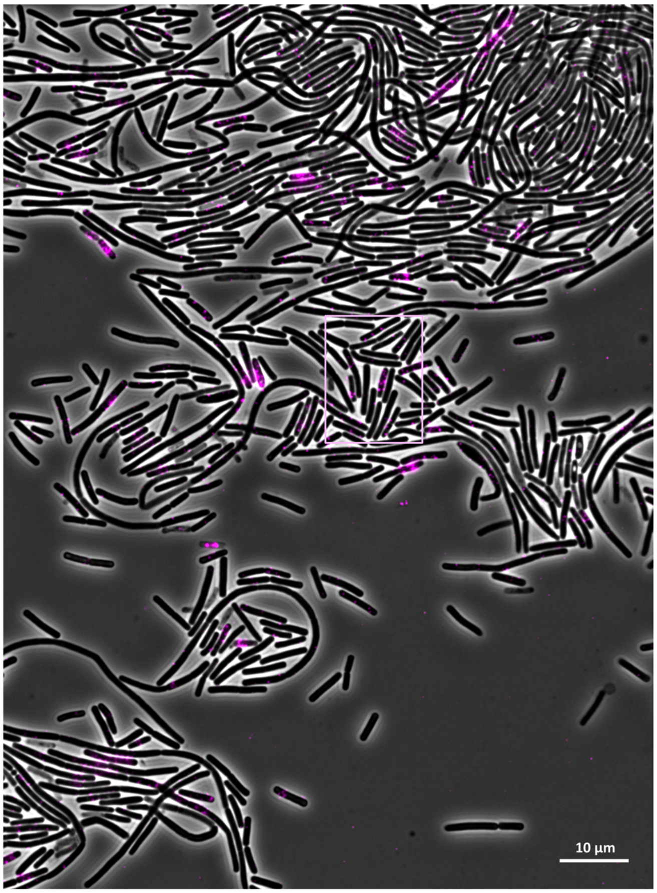

Figure EV1. Tie distribution on the surface of donor cells is heterogeneous.

Image of a large field corresponding to Fig. 5D (inset). Donor cells (SH461: WT/ $pLS20_{cm}$-*tie*$_{\text{-2xHA}}$) were grown to OD_{600} 0.8 and visualized by immunofluorescence microscopy using primary anti-HA antibodies and Alexa647 conjugated secondary antibodies. Cells were not permeabilized before antibody treatment to enable the selective visualization of surface exposed Tie. Shown is an overlay image of phase contrast (gray) with fluorescence from Tie-$_{\text{2xHA}}$ (magenta).

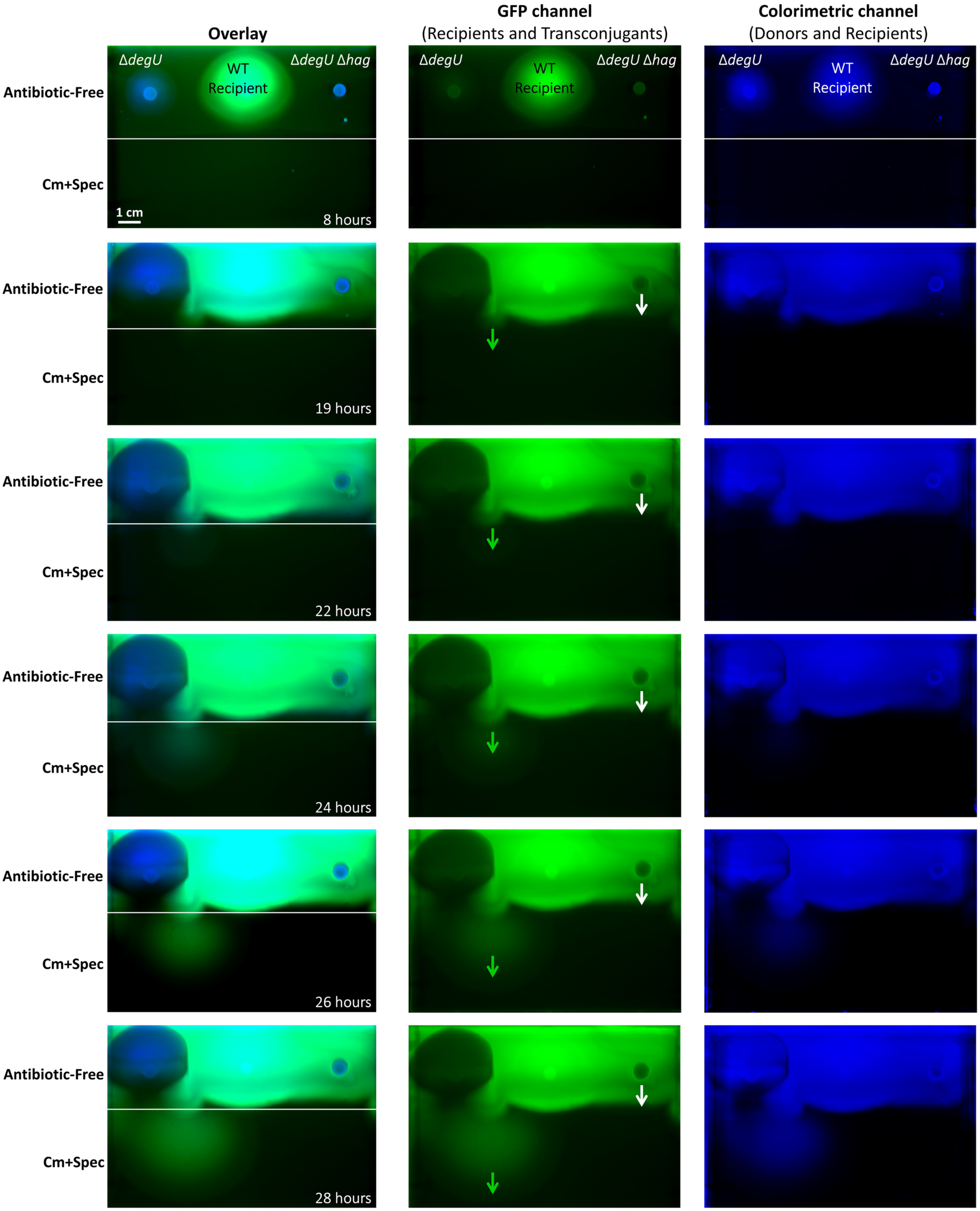
Overlay
GFP channel
(Recipients and Transconjugants)
Colorimetric channel
(Donors and Recipients)
ΔdegU
WT
Recipient
ΔdegU Δhag
Antibiotic-Free
Cm+Spec
1 cm
8 hours
19 hours
22 hours
24 hours
26 hours
28 hours

 © The Author(s)

Figure EV2. Coupling motility and conjugation is beneficial for invading new niches.

Images of entire plates corresponding to the series presented in Fig. 7B (left panels). Bacteria were grown to mid-logarithmic phase and spotted on the antibiotic-free region of the plate. Plates were spotted with Δ*degU* (SH592: Δ*degU*/pLS20$_{cm}$), Δ*hag* Δ*degU* (SH593: Δ*hag* Δ*degU*/pLS20$_{cm}$), and WT (AR16: P_{rrnE}-*gfp*) strains. Presented are overlay images of colorimetric channel (blue) with fluorescence from GFP (green), and their corresponding separate channels, captured at the indicated time points post incubation using ChemiDoc MP imaging system. Shown are full-plate images, including both the antibiotic-free and antibiotic-containing regions (Cm+Spec), with white lines demarcating the border between the two. Arrows indicate the intersection regions of the recipients with the motile (green) and non-motile (white) donors, and the subsequent migration of transconjugants into the selective zone.

© The Author(s)
